# Diarrhoea prevalence in children under five years of age in rural Burundi: an assessment of social and behavioural factors at the household level

**DOI:** 10.3402/gha.v7.24895

**Published:** 2014-08-21

**Authors:** Katharina Diouf, Patrik Tabatabai, Jochen Rudolph, Michael Marx

**Affiliations:** 1 Institute of Public Health, University of Heidelberg, Heidelberg, Germany; 2 Department of Gynaecology and Obstetrics, Heidelberg University Hospital, Heidelberg, Germany; 3 Programme Sectoriel Eau - German Development Cooperation/Deutsche Gesellschaft für Internationale Zusammenarbeit GmbH, Bujumbura, Burundi

**Keywords:** developing countries, diarrhoea, prevention, control, Burundi, water supply, sanitation, hygiene, children

## Abstract

**Background:**

Diarrhoea is the second leading cause of child mortality worldwide. Low- and middle-income countries are particularly burdened with this both preventable and treatable condition. Targeted interventions include the provision of safe water, the use of sanitation facilities and hygiene education, but are implemented with varying local success.

**Objective:**

To determine the prevalence of and factors associated with diarrhoea in children under five years of age in rural Burundi.

**Design:**

A cross-sectional survey was conducted among 551 rural households in northwestern Burundi. Areas of inquiry included 1) socio-demographic information, 2) diarrhoea period prevalence and treatment, 3) behaviour and knowledge, 4) socio-economic indicators, 5) access to water and water chain as well as 6) sanitation and personal/children's hygiene.

**Results:**

A total of 903 children were enrolled. The overall diarrhoea prevalence was 32.6%. Forty-six per cent (n=255) of households collected drinking water from improved water sources and only 3% (n=17) had access to improved sanitation. We found a lower prevalence of diarrhoea in children whose primary caretakers received hygiene education (17.9%), boiled water prior to its utilisation (19.4%) and were aged 40 or older (17.9%). Diarrhoea was associated with factors such as the mother's age being less than 25 and the conviction that diarrhoea could not be prevented. No gender differences were detected regarding diarrhoea prevalence or the caretaker's decision to treat.

**Conclusions:**

Diarrhoea prevalence can be reduced through hygiene education and point-of use household water treatment such as boiling. In order to maximise the impact on children's health in the given rural setting, future interventions must assure systematic and regular hygiene education at the household and community level.

In 2011, diarrhoea accounted for 700,000 deaths in children under five years of age (U5s) worldwide making it the second leading cause of child mortality (1). The highest rates of child mortality are in Sub-Saharan Africa and Southeast Asia. An estimated 1.7 billion episodes of diarrhoea, equalling approximately 2.9 episodes per child per year, created health system costs of about 7 billion US dollars (2). The majority of diarrhoeal diseases can be prevented by implementing water, sanitation and hygiene (WASH) programmes, which all aim at interrupting faecal–oral transmission pathways, commonly referred to as the five “F” (fluids, fields, flies, fingers and food) (3). Several studies have attempted to evaluate the effects of combined or single water, hygiene and sanitation interventions on diarrhoea as an outcome variable. In her review, Fewtrell et al. (4) provided valuable information about the effect of WASH interventions on diarrhoea prevalence, updating an earlier review by Esrey et al. (5). Preventing diarrheal diseases and associated morbidity in children was shown to reduce levels of pupil absence in schools, while preventing long-term consequences such as malnutrition and stunting, which in turn detract from intellectual capacities and later economic status (6). Even though diarrhoea morbidity and mortality has decreased since the 1990s, the overall disease burden remains unacceptably high, particularly in low- and middle-income countries. To achieve sustainable progress in overcoming such unmet health needs, programme planning and implementation needs to be adjusted to the specific requirements and needs of a local setting (7).

## The local context

Burundi is populated by an estimated 9.85 million people and covers an area of 27,834 km^2^, ranking it among both the smallest and most densely populated countries in the African Great Lakes region of Southeast Africa. The majority (89%) of Burundians reside in rural areas. The civil war between 1993 and 2003 left the country's health care and economy deeply shattered. The 2011 Human Development Index ranked Burundi on place 185 out of 187 countries (8).

According to national health centre data, diarrhoea is the third leading cause of morbidity among children under five and the fourth leading cause of mortality in Burundi (9). In 2010, the Burundian Demographic and Health Survey (DHS) reported that 25% of children under five in Burundi had an episode of diarrhoea within the past two weeks (10). During the past years, factors influencing diarrhoea prevalence in Burundi were largely neglected by the research community, with few exceptions (11, 12). This in turn hinders informed decision making as well as effective health planning and programme implementation. Therefore, we set out to define the current diarrhoea prevalence among U5s in rural Burundi and to identify social and behavioural factors associated with the condition.

## Materials and methods

### Study setting

The study was conducted in the province of Bubanza in northwestern Burundi and included two neighbouring settlements (Buhurika and Gatura), which are populated by an estimated 4,746 people (13). The two settlements were selected in order to compare the influence of improved or unimproved water sources on diarrhoea prevalence. Buhurika's population had access to improved water sources, whereas the population of Gatura did not. These settlements were chosen since they represented a project area of the Deutsche Gesellschaft für Internationale Zusammenarbeit (GIZ) GmbH. In June 2010, the Water Sector Programme “PROSECEAU” of the GIZ implemented a water supply project in Buhurika. A total of 33 water taps and 30 private water supplies were set-up or renovated (14). Prior to this intervention, the theoretical access to improved water sources in this settlement was 0% (13).

Other criteria such as landscape, culture and living conditions, socio-economic status, educational level of the population and number of children were similar in both settlements.

### The household survey

**Fig. 1:**
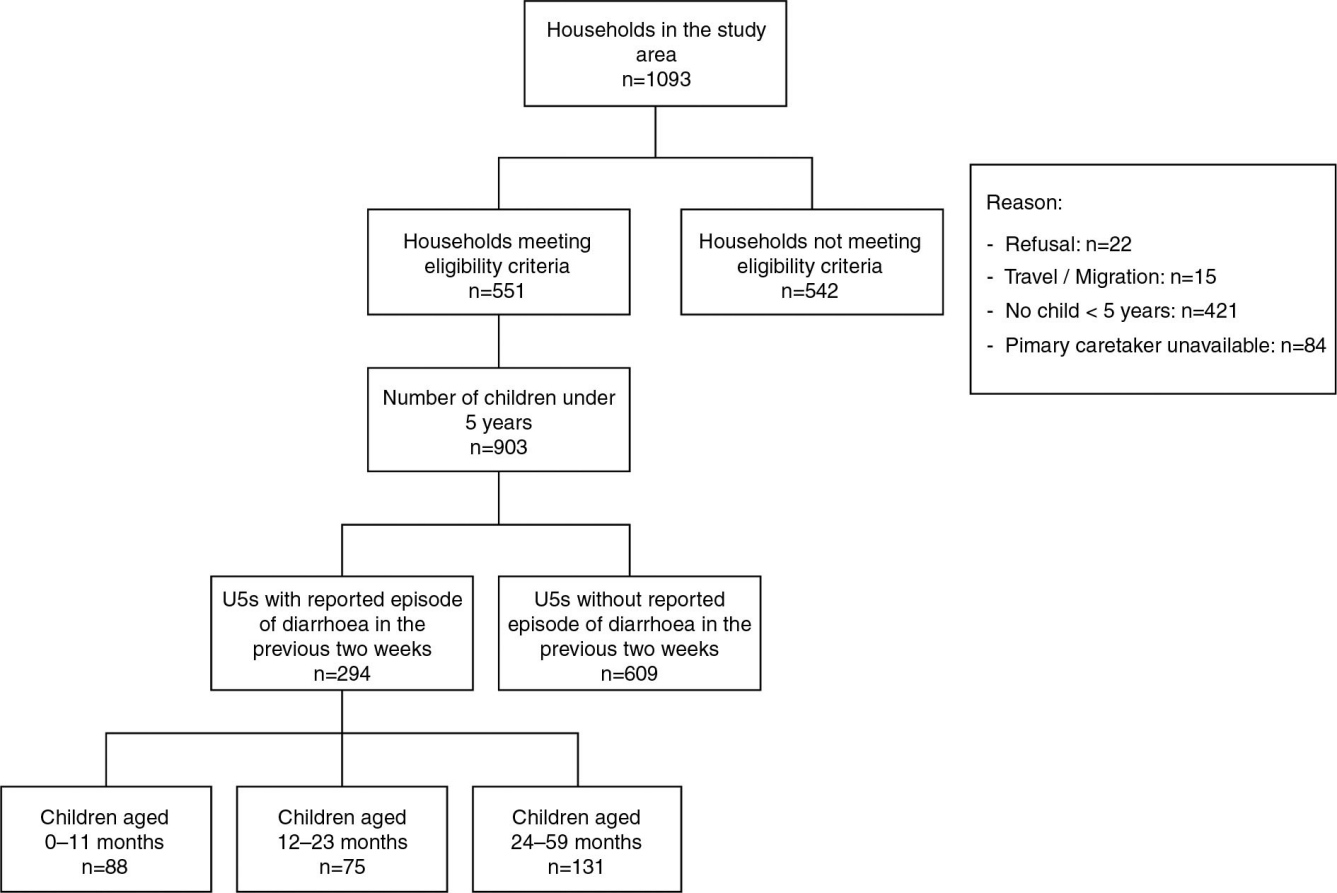
Summary of household survey. Total number of children aged 0–11 months: n=208; 12–23 months: n=157; 24–59 months: n=538.

A total of 551 households were surveyed in the study area (period June–July 2011; Fig. 1). Households qualified for inclusion in the survey if the following criteria were met: 1) primary caretaker was available for interview (mostly the mother) and 2) at least one U5 was living in the household. Three female Burundian research assistants using a paper-based questionnaire conducted interviews in the national language Kirundi. Female interviewers were selected to obtain a woman-to-woman interaction, which was thought to achieve higher authenticity of the information reported (15). Interviewers observed and recorded the living conditions of each household (e.g. type and condition of toilets, recipient for water storage) on site.

The questionnaire was adapted from WHO's and UNICEF's “Core questions on drinking-water and sanitation for household surveys” (16) and covered for the following topics: 1) socio-demographic information, 2) diarrhoea occurrence and treatment, 3) behaviour and knowledge, 4) socio-economic indicators, 5) water access and water chain and 6) sanitation, personal and children's hygiene. Diarrhoeal prevalence among U5s was calculated using the number of children who reportedly had at least one episode of diarrhoea within the preceding two weeks of the survey as numerator and the overall number of U5s as denominator (period prevalence). In accordance to the WHO, diarrhoea was defined as the passage of three or more loose or liquid stools within 24 hours. The presence of blood in the stool, suggesting dysentery, was also considered as diarrhoea (17). To evaluate factors influencing diarrhoea prevalence, 117 indicators were developed on the basis of acknowledged literature in the field (18–22), as well as in depth discussions with local authorities.

The survey questionnaire was pre-tested and standard operational procedures were developed and discussed in-depth with the conducting Burundian research assistants during preparatory workshops.

### Statistical analysis

Statistical analysis was performed using SPSS Statistics Version 20 and included Chi-square testing of all indicators. Variables that reached a Likelihood ratio p-value <0.1 were included in a generalised estimated equation (GEE) logistic regression analysis. Besides, other variables with a scientifically proven impact on diarrhoea were included in the model (23). The dependence of observations within households (more than one U5 per household) was taken into account. The level of significance was set to 0.05 and 95% confidence intervals (CI) were used throughout. All shown p-values were exploratory. Therefore, no adjustments for multiple testing were done.

Descriptive statistics included means, ranges and percentages, as appropriate. In order to detect differences in diarrhoea prevalence based on age, three age strata were formed.

### Ethics and informed consent

Ethical clearance for this study was granted by the Ethical Review Board at the University of Heidelberg Germany (study protocol number: S-629/2011). The study was conducted on behalf of the GIZ with the consent of the Burundian Ministry for Public Health. As of yet, Burundi does not possess a formally registered ethical review board. Verbal informed consent was obtained from all respondents prior to the interview.

## Results

### Socio-demographic characteristics

Table 1 provides an overview of the socio-demographic characteristics of the households surveyed. Most caretakers (51.7%; n=280) were between 20 and 29 years old and had one to two children under the age of five (90.7%; n=500). Over 46% (n=254) of households had at least one U5 who suffered from diarrhoea during the past two weeks. The classification of water sources into improved vs. unimproved revealed that 46.3% (n=255) of households collected drinking water from improved water sources. Public taps were the most common source of drinking water (29.4%; n=162). The classification of excrement disposal facilities into improved vs. unimproved showed that 3.1% (n=17) of households had access to improved sanitation facilities. The majority of households (93.1%; n=513) used open pit latrines as sanitation facilities.

**Table 1:** Socio-demographic data of 551 households surveyed^a^

	Buhurika (N=186) n (%)^b^	Gatura (N=365) n (%)^b^	Overall (N=551) n (%)^b^
**Caretaker characteristics**			
Age in years			
18–19	6 (3.3)	23 (6.4)	29 (5.4)
20–24	43 (23.6)	101 (28.1)	144 (26.6)
25–29	37 (20.3)	99 (27.5)	136 (25.1)
30–34	41 (22.5)	55 (15.3)	96 (17.7)
35–39	32 (17.6)	28 (7.8)	60 (11.1)
40+	23 (12.6)	54 (15.0)	77 (14.2)
Illiteracy	106 (57.0)	245 (67.1)	351 (63.7)
**Household characteristics/economic standing**			
Number of household members			
2–4	66 (35.5)	159 (43.6)	225 (40.8)
5–7	91 (48.9)	160 (43.8)	251 (45.6)
8–10	25 (13.4)	43 (11.8)	68 (12.3)
10+	4 (2.2)	3 (0.8)	7 (1.3)
Number of children <5 years			
1	85 (45.7)	167 (45,8)	252 (45.7)
2	87 (46.8)	161 (44.1)	248 (45.0)
3	14 (7.5)	35 (9.6)	49 (8.9)
4	0	2 (0,5)	2 (0.4)
One or more children <5 suffering from diarrhoea	84 (54.2)	170 (46.6)	254 (46.1)
House made of bricks	150 (80.6)	204 (55.9)	354 (64.2)
Source of income other than agriculture	16 (8.6)	18 (4.9)	34 (6.2)
Self-reported lack of money	26 (14.0)	51 (14.0)	77 (14.0)
**Facilities**			
Hand washing facility	18 (9.7)	27 (7.4)	45 (8.2)
Kitchen	77 (41.4)	125 (34.2)	202 (36.7)
Drinking water source			
Public tab/stand pipe	150 (80.6)	12 (3.3)	162 (29.4)
Private supply	2 (1.1)	4 (1.1)	6 (1.1)
Protected source	27 (14.5)	48 (13.2)	75 (13.6)
Unprotected source	1 (0.5)	3 (0.8)	4 (0.7)
Protected borehole/tube well	1 (0.5)	11 (3.0)	12 (2.2)
River/other surface water	5 (2.7)	287 (78.6)	292 (53.0)
Non-drinking water source			
Public tab/Stand pipe	88 (47.3)	7 (1.9)	95 (17.2)
Private supply	0	4 (1.1)	4 (0.7)
Protected source	15 (8.1)	28 (7.7)	43 (7.8)
Unprotected source	7 (3.8)	13 (3.6)	20 (3.6)
Borehole/tube well	5 (2.7)	35 (9.6)	40 (7.3)
River/other surface water	69 (37.1)	278 (76.2)	347 (63.0)
Drinking water collection time			
<10 min	58 (31.2)	52 (14.2)	110 (20.0)
10–30 min	76 (40.9)	124 (34.0)	200 (36.3)
>30 min	52 (28.0)	189 (51.8)	241 (43.7)
Toilet			
Flush or pour flush	0	2 (0.5)	2 (0.4)
Pit latrine with slab	5 (2.7)	10 (2.7)	15 (2.7)
Pit latrine without slab (open pit)	169 (90.9)	344 (94.2)	513 (93.1)
Hole	12 (6.5)	8 (2.2)	20 (3.6)
Open defecation	0	1 (0.3)	1 (0.2)
Toilet shared with other households	54 (31.0)	132 (37.6)	186 (33.8)
**Behavioural characteristics**			
Boiling water before consumption	8 (4.3)	13 (3.6)	21 (3.8)
Litres of water used per capita per day^c^			
1 to <5	21 (11.3)	18 (4.9)	39 (7.1)
5 to <10	86 (46.2)	175 (47.9)	261 (47.4)
10 to <15	56 (30.1)	136 (37.3)	192 (34.8)
15+	23 (12.4)	36 (9.9)	59 (10.7)

a Data were missing for fewer than 2% of participants for all responses.

b Frequencies and percentages are unweighted.

c During dry season.

It is of note that only five households had access to improved sanitation facilities as well as improved water sources simultaneously. Only one primary caretaker named all of WHO's critical times of hand washing correctly when asked at what moments during day she washes her hands.

### Gender, age and diarrhoea

**Fig. 2:**
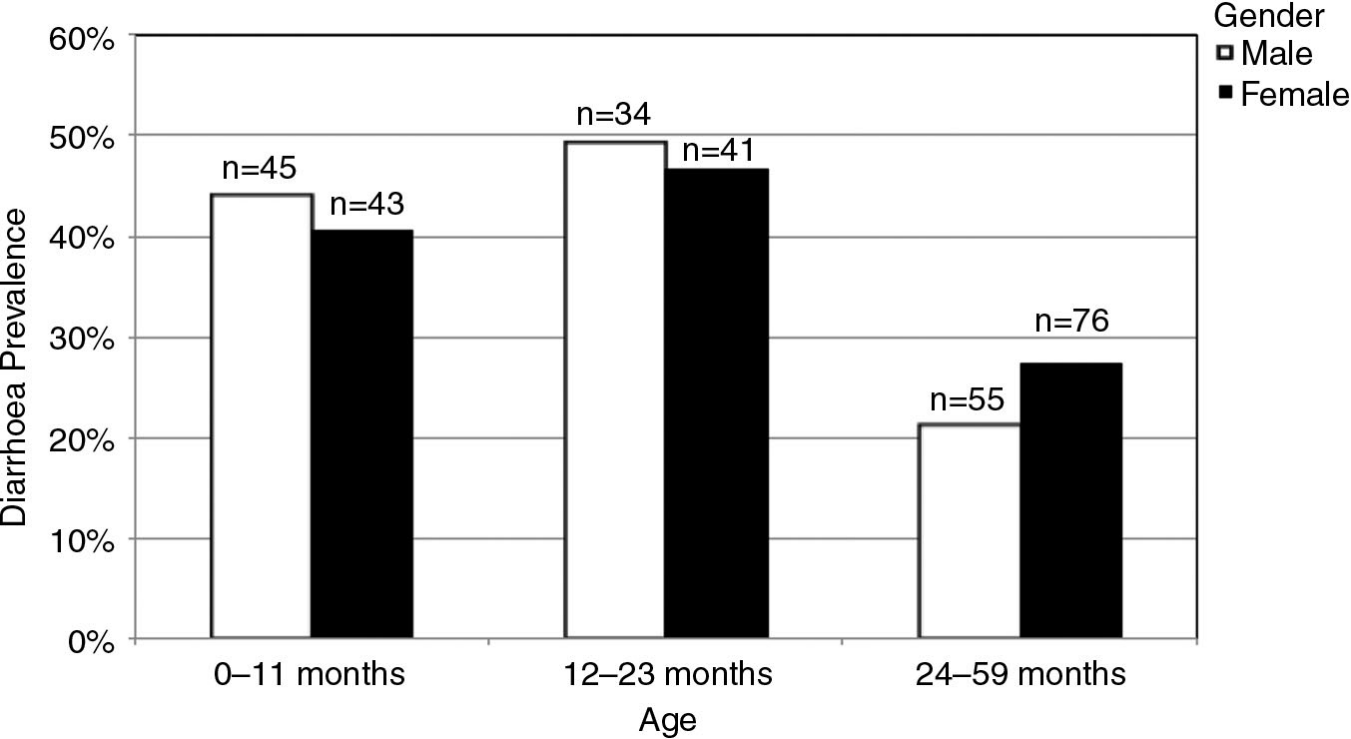
Diarrhoea prevalence per age group and gender (N=294 children).

**Fig. 3:**
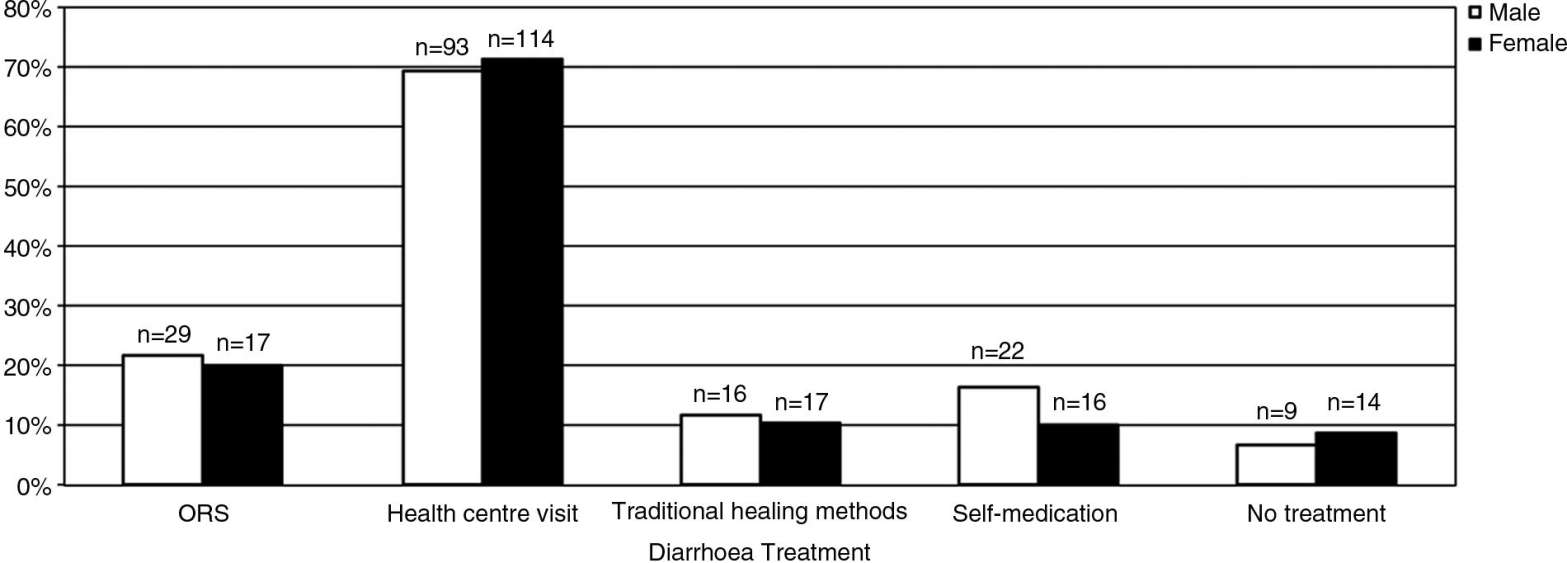
Diarrhoea treatment choices depending on children's gender.

The analysis per age strata revealed a higher prevalence of diarrhoea in the first two years of life (Fig. 2). Across age strata, the overall prevalence was at 32.6% (n=294). Gender did not play a substantial role regarding diarrhoea prevalence across the different age groups. An analysis of treatment options administered to boys and girls during their last diarrhoeal episode such as the use of oral rehydration salts (ORS), traditional healing methods, self-medication, the visit of a health centre or no treatment at all, also discovered no differences between the two genders (Fig. 3).

### Generalised estimated equations

In the GEE analysis (Table 2), we found children whose primary caretakers received hygiene education in the past (OR 0.45; 95% CI: 0.21–0.97; p=0.043), boiled water before consumption (OR 0.39; 95% CI: 0.16–0.98; p=0.044), and were aged 40 or older (OR 0.51; 95% CI: 0.30–0.85; p=0.009) less likely to suffer from diarrhoea.

**Table 2:** Generalised estimated equations with diarrhoea as response variable

Explanatory variable	OR	95% CI	p-value
**Education and socio-demographics**			
Site	0.92	0.65–1.31	0.643
Hygiene education	0.45	0.21–0.97	0.043
Hand washing as prevention method against diarrhoea	0.66	0.41–1.05	0.081
“Diarrhoea cannot be prevented”	2.02	1.19–3.44	0.009
“I do not know what causes diarrhoea”	1.17	0.75–1.84	0.492
Age of primary caretaker <25 years	1.40	1.02–1.91	0.035
Age of primary caretaker 40 years and more	0.51	0.30–0.85	0.009
Source of income other than agriculture	0.76	0.38–1.52	0.443
**Water sources and water chain**			
Exclusive use of improved water sources	0.78	0.54–1.15	0.209
Drinking-water collection time less than 10 min	1.62	1.11–2.35	0.012
Clean receptacle to drink from	0.78	0.57–1.05	0.104
**Sanitation and hygiene**			
Access to improved sanitation	1.49	0.62–3.54	0.371
Sanitary disposal of children's faeces	1.03	0.75–1.43	0.854
Proper food hygiene	0.95	0.66–1.37	0.795
Boiling water before consumption	0.39	0.16–0.98	0.044
Proper children's hygiene	0.98	0.72–1.33	0.886

OR=odds ratio; CI=confidence interval.

In contrast, higher rates of diarrhoea prevalence were seen in children whose primary caretaker believed that diarrhoea cannot be prevented (OR 2.02; 95% CI: 1.19–3.44; p=0.009) or was under the age of 25 years (OR 1.40; 95% CI: 1.02–1.91; p=0.035) as well as for a drinking-water collection time less than 10 minutes (OR 1.62; 95% CI: 1.11–2.35; p=0.012).

Access to improved sanitation as well as exclusive use of improved water sources had no significant effect on diarrhoea prevalence in our study.

Table 3 provides detailed information about the indicators included in the GEE analysis.

**Table 3:** Explanation of indicators included in the GEE-model

Indicator	Explanation
**Education and socio-demographics**	
Hygiene education	Mothers who received hygiene education through a health promoter in the past
Hand washing as prevention method against diarrhoea	Primary caretakers who believe diarrhoea can be prevented through hand washing with soap
“Diarrhoea cannot be prevented”	Primary caretakers who believe diarrhoea cannot be prevented
“I do not know what causes diarrhoea”	Primary caretakers who are not able to name a possible cause of diarrhoea
Source of income other than agriculture	Households with at least one family member working as a salesman, bricklayer, potter or officer
**Water sources and water chain**	
Exclusive use of improved water sources	Exclusive use of improved sources such as public taps, private supplies, protected springs, tube wells/boreholes or rainwater collection as sole drinking water source as well as source of water for cooking, washing and personal hygiene
Drinking-water collection time <10 min	Collection time for a round-trip including queuing <10 minutes
Clean receptacle to drink from	Receptacle to drink from stored in a fixed place protected from pollution
**Sanitation and hygiene**	
Access to improved sanitation	Use of available improved sanitation facilities in the study area (pit latrines with slap, flush toilets) by less than 30 persons
Sanitary disposal of children's faeces	Child uses toilet/latrine or faeces are put/rinsed into the toilet/latrine after defecation or faeces are buried
Proper food hygiene	Preparation of meals inside the house in a room designated only for cooking, washing of fruits/vegetables before raw consumption, protection of foods against insects by covering with plates
Proper children's hygiene	Daily washing of children and daily changing of children's clothes

## Discussion

This study provides detailed information about household characteristics in a rural setting of northwestern Burundi and their interrelation with diarrhoea prevalence among the U5 population. Our findings indicate that diarrhoea prevalence at the household level was higher than previously reported (32.6% vs. 28.9%) (10). Likewise, the 2010 Burundian Demographic and Health Survey (DHS) found higher percentages of U5s who received ORS during their last diarrheal episode (38% vs. 20.7%). Opposite to our study findings, higher percentages of rural households with access to improved sanitation and improved water sources were reported (sanitation: 32% vs. 3.1%; water supply: 74% vs. 46%).

Our analysis of possible gender inequities in health-care seeking and choice of treatment for common diarrhoea presented similar results to recent studies from South Asia and Sub-Saharan Africa. These studies showed no differences in care-seeking behaviour or at times even a preference of the female sex (24–26). Nevertheless, there is evidence supporting the assumption that girls experience higher treatment delays (27–29) and are less likely to be examined by a qualified health care provider (30). Besides, the higher prevalence rates of diarrhoea in the first two years of life are analogous to previous findings (31).

The association between the age of the caretaker and disease rates may be explained through an increasing experience in childcare, improving hygiene and feeding practices with advanced age. The results emphasise the protective effect of knowledge in the fight against diarrhoeal diseases, which is achievable through hygiene education. Hygiene promotion is able to avert 200 DALY's per $1,000 spent, making it the most cost-effective public health intervention in the world (32). Fewtrell et al. ascribe hygiene interventions to reduce diarrhoeal diseases by 37%, under which promotion of hand washing with soap compared to general hygiene education showed the strongest effect (4, 33). Similar findings were reported by earlier reviews of Esrey and Huttly et al. (5, 34).

Providing access to improved water supply and sanitation is an important cornerstone in reducing diarrhoeal disease rates. However, these actions must be integrated into a comprehensive approach. In order to improve people's hygiene behaviour and to assure proper utilisation of new facilities, hygiene education at the community and household level is essential (1). Bartram et al. called on health professionals to provide global access to hygiene promotion, especially for young children's parents, in order to improve the current situation (35).

Part of most hygiene education is the provision of information regarding the correct handling of drinking water, such as purifying techniques prior to drinking. Supporters of point-of-use water treatment interventions argue that even water that was clean at source is under high risk of contamination due to unhygienic drawing or storage and thus should be treated directly before consumption. In Burundi, such point-of-use water treatment methods are poorly utilised with “boiling” being the most frequent treatment option (countrywide used by 4% vs. 3.8% in our study setting) (10). Our findings indicate that boiling water before consumption was able to reduce diarrhoea in U5s by 61% in the study area while the sole use of improved sources had no impact on diarrhoea prevalence. There is evidence supporting this finding (4, 36), even if these early enthusiastic results were qualified by later studies (37). Point-of-use water treatment options should not be seen as a replacement for more cost-intensive water supply projects since sustainable development can only be achieved by improving both water quality and quantity. It is important to provide accessible, consistently safe drinking water through water supply projects because the provision of improved sanitation is also linked to these projects in the long term (38). In accordance with the WHO's Guidelines for drinking water quality (39), our findings show that in a remote environment such as rural Burundi where sources of contamination are omnipresent, boiling water before consumption can make a valuable contribution in the fight against diarrhoea.

Another controversial debate, reflected by our study results, addresses the relationship of time to water source (TTWS) and diarrhoeal episodes. Our results were similar to findings from Gascon et al. which showed a higher risk of diarrhoea when TTWS decreased (40). A possible explanation for our finding could be a higher exposure to contaminated drinking water and thus higher chances of pathogen uptake and transmission (40). However, the data currently available does not allow any final conclusions about the relationships of TTWS and diarrhoea (41).

In our study, no link between the utilisation of different water sources or improved sanitation and diarrhoea prevalence was found. Depending on the aetiology, diarrhoeal diseases can be transmitted through a multitude of pathways. Blocking one or two transmission pathways cannot meet the purpose, as sources of infection remain ubiquitous, which may explain the missing effect in our study setting.

We conclude that any intervention aimed to increase knowledge about diarrhoea prevention as well as basic hygiene practices is likely to reduce diarrhoea morbidity in similar rural settings. One year after the implementation of the new water supply system GIZ trained two health promoters to sensitise the population on topics such as water, hygiene, waste water elimination, sanitation and HIV/Aids using the SARAR approach (42). Prior to our study only one session was held to a small subset of the local population. This stepwise implementation represents an excellent opportunity for future research in which results of our study can function as baseline data.

The results of this study have been communicated to GIZ authorities as well as Burundian partners and will be available for future project planning. Modest investment in research with clear and practical goals is likely to be repaid by the increased effectiveness of future interventions as well as cohort studies to better determine the probable transmission pathways of diarrhoeal diseases in this rural area.

### Limitations

Household surveys and observations in general may be subject to courtesy-bias. The missing link between the choice of water source and diarrhoea prevalence might be related to incomplete reporting. People tend to use unsafe water on an irregular basis, e.g. due to rigid source opening hours. However, even sporadic exposure to unsafe water can limit health gains (43). Due to financial and logistic restrictions no microbiological and chemical analysis of drinking water was undertaken at source or household level. The possible consummation of contaminated water cannot be excluded and the assumption that consumed water of improved sources was harmless is based on prior analysis of water quality undertaken by the GIZ in July 2009.

Regarding the selected WASH indicators, it should be noted that these do not comprise a comprehensive list of all elements influencing diarrhoeal diseases.

## Conclusion

Our study shows that existing baseline data regarding diarrhoea in rural Burundi underestimated the disease burden. Awareness training concerning the use of ORS in such rural areas should be increased and existing campaigns changed to the needs of the local population. Gender inequities in care seeking behaviour seem to play a secondary role in the provision of good treatment. The results of this study can be useful in the development of future interventions aiming to reduce diarrhoea occurrence in similar settings. Amongst others, our study outlines how diarrhoea prevalence can be positively influenced by improving basic hygiene practices and the knowledge about this disease. Future projects should include a profound hygiene education of the local population in order to maximise the impact on children's health.

## References

[1] Bhutta ZA, Das JK, Walker N, Rizvi A, Campbell H, Rudan I (2013). Interventions to address deaths from childhood pneumonia and diarrhoea equitably: what works and at what cost?. Lancet.

[2] Hutton G, Haller L (2004). Evaluation of the costs and benefits of water and sanitation improvements at the global level.

[3] Wagner EG, Lanoix JN (1958). Excreta disposal for rural areas and small communities. Monogr Ser World Health Organ.

[4] Fewtrell L, Kaufmann RB, Kay D, Enanoria W, Haller L, Colford JM (2005). Water, sanitation, and hygiene interventions to reduce diarrhoea in less developed countries: a systematic review and meta-analysis. Lancet Infect Dis.

[5] Esrey SA, Potash JB, Roberts L, Shiff C (1991). Effects of improved water supply and sanitation on ascariasis, diarrhoea, dracunculiasis, hookworm infection, schistosomiasis, and trachoma. Bull World Health Organ.

[6] Walker SP, Wachs TD, Grantham-McGregor S, Black MM, Nelson CA (2011). Inequality in early childhood: risk and protective factors for early child development. Lancet.

[7] IOM (2009). Global issues in water, sanitation, and health. http://www.ncbi.nlm.nih.gov/books/NBK28462/.

[8] UNDP (2011). Human development report 2011–sustainability and equity: a better future for all. http://www.undp.org/content/undp/en/home/librarypage/hdr/human_developmentreport2011.html.

[9] Republic of Burundi, Ministry of Public Health, National Epidemiology and Health Statistics Department (EPISTAT)

[10] Institut de Statistiques et d’Études Économiques du Burundi (ISTEEBU), Ministère de la Santé Publique et de la Lutte contre le Sida [Burundi] (MSPLS), ICF International (2012). Enquête Démographique et de Santé Burundi 2010.

[11] Birmingham ME, Lee LA, Ntakibirora M, Bizimana F, Deming MS (1997). A household survey of dysentery in Burundi: implications for the current pandemic in sub-Saharan Africa. Bull World Health Organ.

[12] Esrey SA (1996). Water, waste, and well-being: a multicountry study. Am J Epidemiol.

[13] Ministry of Energy and Mines [Burundi], Deutsche Gesellschaft für Internationale Zusammenarbeit (GIZ) (2007). INEA Atlas-Province de Bubanza.

[14] Deutsche Gesellschaft für Internationale Zusammenarbeit, Programme Sectoriel Eau (ProSecEau). http://www.giz.de/en/worldwide/19214.html.

[15] Samanta BB, Van Wijk CA (1998). Criteria for successful sanitation programmes in low income countries. Health Policy Plan.

[16] World Health Organization, United Nations Children's Fund (2006). Core questions on drinking-water and sanitation for household surveys. http://www.who.int/water_sanitation_health/monitoring/oms_brochure_core_questionsfinal24608.pdf.

[17] WHO http://www.who.int/entity/mediacentre/factsheets/fs330/en/index.html.

[18] Billig P, Bendahmane D, Swindale A (1999). Water and sanitation indicators measurement guide.

[19] Child Survival and Technical Support Project, United States Agency for International Development, Child Survival Collaborations and Resources Group (2000). Knowledge, practices and coverage survey. http://pdf.usaid.gov/pdf_docs/PNACK209.pdf.

[20] UNICEF (1995). Monitoring progress toward the goals of the world summit for children- a practical handbook for multiple-indicator surveys.

[21] Howard G, Bartram J (2003). Domestic water quantity, service level and health. http://www.who.int/water_sanitation_health/diseases/WSH03.02.pdf.

[22] WHO, UNICEF (2013). Joint monitoring programme for water supply and sanitation. Progress on drinking water and sanitation. http://www.who.int/water_sanitation_health/publications/2013/jmp_report/en/.

[23] Emina JB, Kandala NB (2012). Accounting for recent trends in the prevalence of diarrhoea in the Democratic Republic of Congo (DRC): results from consecutive cross-sectional surveys. BMJ Open.

[24] Nasrin D, Wu Y, Blackwelder WC, Farag TH, Saha D, Sow SO (2013). Health care seeking for childhood diarrhea in developing countries: evidence from seven sites in Africa and Asia. Am J Trop Med Hyg.

[25] Diaz T, George AS, Rao SR, Bangura PS, Baimba JB, McMahon SA (2013). Healthcare seeking for diarrhoea, malaria and pneumonia among children in four poor rural districts in Sierra Leone in the context of free health care: results of a cross-sectional survey. BMC Public Health.

[26] Das SK, Nasrin D, Ahmed S, Wu Y, Ferdous F, Farzana FD (2013). Health care-seeking behavior for childhood diarrhea in Mirzapur, rural Bangladesh. Am J Trop Med Hyg.

[27] Malhotra N, Upadhyay RP (2013). Why are there delays in seeking treatment for childhood diarrhoea in India?. Acta Paediatr.

[28] Willis JR, Kumar V, Mohanty S, Singh P, Singh V, Baqui AH (2009). Gender differences in perception and care-seeking for illness of newborns in rural Uttar Pradesh, India. J Health Popul Nutr.

[29] Wilson SE, Ouedraogo CT, Prince L, Ouedraogo A, Hess SY, Rouamb N (2012). Caregiver recognition of childhood diarrhea, care seeking behaviors and home treatment practices in rural Burkina Faso: a cross-sectional survey. PLoS One.

[30] Najnin N, Bennett CM, Luby SP (2011). Inequalities in care-seeking for febrile illness of under-five children in urban Dhaka, Bangladesh. J Health Popul Nutr.

[31] WHO, UNICEF (2009). Diarrhoea: why children are still dying and what can be done. http://www.who.int/maternal_child_adolescent/documents/9789241598415/en/.

[32] Laxminarayan R, Chow J, Shahid-Salles SA, Jamison DT, Breman JG, Measham AR, Alleyne G, Claeson M (2006). Intervention cost-effectiveness: overview of main messages. Disease control priorities in developing countries.

[33] Curtis V, Cairncross S (2003). Effect of washing hands with soap on diarrhoea risk in the community: a systematic review. Lancet Infect Dis.

[34] Huttly SR, Morris SS, Pisani V (1997). Prevention of diarrhoea in young children in developing countries. Bull World Health Organ.

[35] Bartram J, Cairncross S (2010). Hygiene, sanitation, and water: forgotten foundations of health. PLoS Med.

[36] Waddington H, Snilstveit B, White H, Fewtrell L (2009). Water, sanitation and hygiene interventions to combat childhood diarrhoea in developing countries.

[37] Cairncross S, Hunt C, Boisson S, Bostoen K, Curtis V (2010). Water, sanitation and hygiene for the prevention of diarrhoea. Int J Epidemiol.

[38] Hunter PR, MacDonald AM, Carter RC (2010). Water supply and health. PLoS Med.

[39] World Health Organization (2011). Guidelines for drinking-water quality. http://www.who.int/water_sanitation_health/publications/2011/dwq_guidelines/en/.

[40] Gascon J, Vargas M, Schellenberg D, Urassa H, Casals C, Kahigwa E (2000). Diarrhea in children under 5 years of age from Ifakara, Tanzania: a case-control study. J Clin Microbiol.

[41] Subaiya S, Cairncross S (2011). Response to wang and hunter: a systematic review and meta-analysis of the association between self-reported diarrheal disease and distance from home to water source. Am J Trop Med Hyg.

[42] Narayan D (1996). Toward participatory research. Technical Paper Number 307.

[43] Hunter PR, Zmirou-Navier D, Hartemann P (2009). Estimating the impact on health of poor reliability of drinking water interventions in developing countries. Sci Total Environ.

